# Clinical Importance of the Inflammatory Prognostic Index in Patients with Sepsis

**DOI:** 10.3390/medicina62061029

**Published:** 2026-05-26

**Authors:** Tugba Bingol Tanriverdi, Veysi Yazar, Ramazan Aslanparcasi, Abdullah Sengul, Zeliha Ayhan, Mahmut Alp Karahan

**Affiliations:** Department of Anesthesiology and Reanimation, Mehmet Akif Inan Training and Research Hospital, University of Health Science, Sanliurfa 63040, Turkey; yazar_2202@hotmail.com (V.Y.); rmzn.aslan.2015@gmail.com (R.A.); abdullahsengul342@gmail.com (A.S.); zelihayhan@hotmail.com (Z.A.); mahmutalp_k@yahoo.com (M.A.K.)

**Keywords:** sepsis, in-hospital mortality, inflammatory markers, inflammatory prognostic index

## Abstract

*Background and Objectives*: Sepsis is a life-threating organ dysfunction condition caused by the body’s uncontrolled response to an infection. Many traditional and novel inflammatory markers have been used to determine poor prognosis in patients’ sepsis. The inflammatory prognostic index (IPI) is also a novel marker of inflammation. As there are no studies examining the association of the IPI with in-hospital mortality in patients with sepsis nor its performance compared with other inflammatory markers, we aimed to investigate the clinical importance of the IPI for predicting in-hospital mortality in sepsis patients. *Materials and Methods*: A total of 157 consecutive patients diagnosed with sepsis were retrospectively included in this study. The systemic immune-inflammation index (SII; platelet × neutrophil/lymphocyte), the systemic inflammatory response index (SIRI; neutrophil × monocyte/lymphocyte), the aggregate index of systemic inflammation (AISI; neutrophil × platelet × monocyte/lymphocyte) and the IPI (C-reactive protein × neutrophil-to-lymphocyte ratio [NLR]/albumin) were calculated for all patients. Patients were divided into two groups: survivors (n = 81) and non-survivors (n = 76). *Results*: Non-survivor patients had significantly higher SII (*p* = 0.002), SIRI (*p* < 0.001), AISI (*p* = 0.002) and IPI (*p* < 0.001) than survivors. The AUC of the IPI was significantly higher than those of the SII (0.751 vs. 0.645; *p* = 0.010) and the AISI (0.751 vs. 0.648; *p* = 0.021) and tended to be higher than that of the SIRI (0.751 vs. 0.687; *p* = 0.091). Based on logistic regression analysis, the IPI was found to be an independent predictor of mortality (OR: 1.007, 95%CI: 1.002–1.012, *p* = 0.006). *Conclusions*: The IPI is a novel combined inflammatory marker that can be easily obtained from laboratory parameters. We determined that the IPI had moderately higher discriminatory ability than the SII and the AISI in patients with sepsis, which indicates that it may be used for risk stratification in this population.

## 1. Introduction

Sepsis is defined as a life-threating organ dysfunction condition caused by the body’s uncontrolled response to an infection. It is a critical process that can lead to fatal outcomes if not treated promptly [1,2]. Despite medical advances, sepsis continues to be a significant global health problem with high morbidity and mortality rates. It is estimated to affect millions of people worldwide each year and cause one in every five deaths globally [2,3]. One of the fundamental principles for the appropriate management of patients with sepsis is the early and accurate identification of patients at high risk of mortality [4,5]. Therefore, simple, bedside-usable and easily obtainable biomarkers are needed for risk stratification in this population.

Sepsis is quite a complex process, and effective patient management is very important to improve outcomes. Therefore, identifying high-risk patients who may have poor outcomes and initiating appropriate therapeutic measures are of paramount importance. Inflammation plays crucial roles in the onset and progression of sepsis [6]. To date, many inflammatory markers, including whole-blood cell-derived inflammatory markers, have been used to determine poor prognosis and evaluate treatment response in high-risk sepsis patients [6,7,8]. Recently, the use of novel combined indices, such as the systemic immune-inflammation index (SII), the systemic inflammatory response index (SIRI), the aggregate index of systemic inflammation (AISI) and the inflammatory prognostic index (IPI), has been introduced into clinical use to assess inflammation [9,10,11,12]. The SII, the SIRI and the AISI are derived from neutrophil, lymphocyte, monocyte and platelet counts, which can be easily obtained from a complete blood count; studies have reported that these indices were associated with prognosis in patients with sepsis [8,11,13]. On the other hand, the IPI is an inflammatory parameter consisting of biochemical C-reactive protein (CRP) and albumin in addition to the neutrophil and lymphocyte counts obtained in the hemogram. Albumin is a negative acute-phase protein and decreases in response to chronic inflammation, whereas CRP is a highly sensitive marker of active inflammation and increases in response to acute inflammation. No studies have investigated whether the combination of neutrophil-to-lymphocyte ratio [NLR] with CRP and albumin, namely, the IPI, may be a better marker than the SII, the SIRI and the AISI.

Although the clinical roles of the SII, the SIRI and the AISI are known in patients with sepsis, there are no studies demonstrating the clinical significance of the IPI in this population. In this study, we aimed to assess the prognostic significance of the IPI in patients with sepsis and compare its clinical value with that of the AISI, the SII and the SIRI in predicting in-hospital mortality.

## 2. Materials and Methods

A total of 157 consecutive patients diagnosed with sepsis and followed up in the intensive care unit of our clinic between January 2020 and July 2024 were retrospectively included in this study. The study protocol was approved by the Harran University Clinical Research Ethics Committee (HRÜ/24.16.19, date: 21 October 2024) and was conducted in accordance with the Declaration of Helsinki. Since the study was retrospective, informed consent was not obtained from the patients. All patients over the age of 18 years whose medical records and laboratory data were available were included. Exclusion criteria were as follows: patients of age less than 18 years, who were pregnant or breastfeeding, and who presented with known inflammatory disease, autoimmune disease, hematologic disorder or malignancies.

The diagnosis of sepsis was based on the Sepsis-3 criteria, according to which sepsis is defined as an acute increase in Sequential Organ Failure Assessment score of ≥2 points from baseline in a patient with suspected infection [5]. The Glasgow Coma Scale (GCS) was used to evaluate the level of consciousness on admission. The demographic information, laboratory results, clinical management and in-hospital outcomes of the patients were recorded from the hospital information management system and patient files. Patients were divided into two groups based on in-hospital mortality, survivors (group 1, n = 81) and non-survivors (group 2, n = 76), and comparisons were made between the groups.

All patients’ blood samples were examined upon admission, and their hemograms and biochemical parameters were recorded, including white blood cell and its subtypes, hemoglobin, platelet, glucose, creatinine, albumin and blood gas analysis. The SII was calculated as platelet count × neutrophil count/lymphocyte count [9]. The SIRI was calculated as neutrophil count × monocyte count/lymphocyte count [9]. The AISI was calculated as neutrophil count × platelet count × monocyte count/lymphocyte count [9]. The IPI was calculated as C-reactive protein (CRP) × NLR/albumin [12].

### Statistical Analysis

SPSS 22.0 was used for data analysis. The Kolmogorov–Smirnov test was used to assess if the continuous data had a normal distribution; if so, they were recorded as means ± SDs and compared using the independent sample *t*-test, while continuous data without a normal distribution were recorded as medians (Q1–Q3) and compared using the Mann–Whitney U test. The Person or Spearman correlation coefficient was used for correlation analysis. The areas under the curves (AUCs) of the IPI, the AISI, the SII and the SIRI for predicting in-hospital mortality were obtained using receiver operating characteristic (ROC) curve analysis. The comparison of the AUCs of the four indices was performed with MedCalc 16 statistic software, trial version, and statistical comparisons of the AUCs were adjusted for multiple testing (c statistics and Delong test). Multivariate logistic regression analysis was used to determine independent predictors of in-hospital mortality. Goodness-of-fit tests (Hosmer–Lemeshow test, Likelihood ratio test, and Chi-square test) and collinearity diagnostic tests (variance Inflation Factor [VIF] and tolerance value) were performed to determine the fit of the regression model, and the Nagelkerke R^2^ in the final step was 0.260. A *p*-value of <0.05 was considered statistically significant.

## 3. Results

Among the 157 patients included in the study, in-hospital mortality was recorded in 76 (48.4%). The median age of the study population was 66 (47–75) years. A comparison of the baseline characteristics of survivors and non-survivors is shown in Table 1. The GCS score at admission was significantly lower in non-survivors compared with survivors (12 [10–15] vs. 15 [13–15]; *p* < 0.001). However, no significant differences in other basal characteristics were found between the two groups (Table 1).

A comparison of the baseline laboratory characteristics of the patients is shown in Table 2. Compared with survivors, non-survivors had significantly higher WBC (*p* = 0.045), neutrophil (*p* = 0.014) and CRP (*p* < 0.001) values but lower lymphocyte count (*p* = 0.002). In addition, it was found that the SII (*p* = 0.002), the SIRI (*p* < 0.001), the AISI (*p* = 0.002) and the IPI (*p* < 0.001) were significantly higher in non-survivors (Table 2).

**Figure 1:**
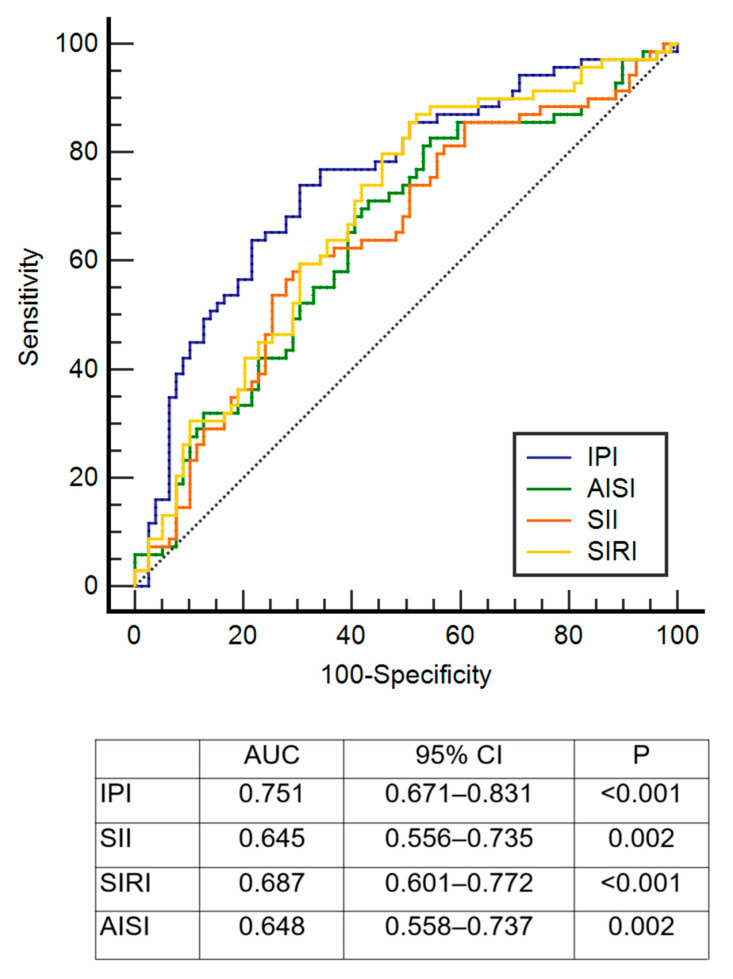
Receiver operating characteristic (ROC) curves of inflammatory parameters for predicting in-hospital mortality.

**Figure 2:**
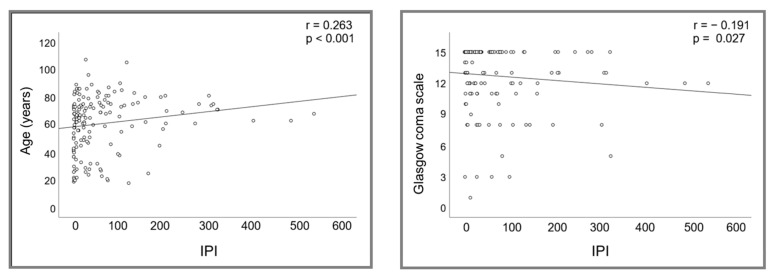
Correlation analysis of IPI with age and Glasgow Coma Scale.

Based on ROC curve analysis, we obtained the AUCs of the SII, the SIRI, the AISI and the IPI for predicting in-hospital mortality, as shown in Figure 1. The AUC of the IPI was significantly higher than those of the SII (0.751 vs. 0.645; 95% CI: 0.026–0.185, *p* = 0.010) and the AISI (0.751 vs. 0.648; 95% CI: 0.016–0.191, *p* = 0.021) and tended to be higher than that of the SIRI (0.751 vs. 0.687; 95% CI: −0.010–0.139, *p* = 0.091) (Figure 1). An IPI ≥ 26.7 predicted in-hospital mortality with a sensitivity of 71%, a specificity of 70%, a positive predictive value of 69% and a negative predictive value of 72.2% (Youden index, J: 0.41). In the correlation analysis, the IPI was found to be positively correlated with age (r = 0.263, *p* < 0.001) but negatively correlated with admission GCS (r= −0.191, *p* = 0.027) (Figure 2).

Using logistic regression analysis, we found that admission GCS (OR: 0.764, 95%CI: 0.659–0.885, *p* < 0.001) and the IPI (OR: 1.007, 95%CI: 1.002–1.012, *p* = 0.006) were independent predictors of in-hospital mortality (Nagelkerke R^2^ in the final step: 0.260) (Table 3).

## 4. Discussion

In this study, we investigated the prognostic value of four indices in patients with sepsis. The main findings of our study are as follows: (I) the SII, the SIRI, the AISI and the IPI were significantly higher in non-survivor sepsis patients than in survivors; (II) the diagnostic ability of the IPI to predict in-hospital mortality was higher than that of the other three combined inflammatory markers; (III) the IPI was determined to be an independent predictor of in-hospital mortality. According to our results, we suggest that the IPI may serve as a beneficial prognostic marker in patients with sepsis.

Sepsis is a dysregulated host response to an infectious agent where a cascade of events is triggered by innate and adaptive immune responses, various cell types are activated, and many pro-inflammatory and anti-inflammatory molecules are released. Each of these makers plays a critical role in the body response to sepsis [14]. Neutrophils are among the most important cells for host defense, as they are the first cells to migrate to the site of infection and help clear the body of the pathogen by releasing pro-inflammatory cytokines. However, they exhibit dysregulated activity in sepsis and may be activated excessively, which provides an additional contribution to the pathogenesis of sepsis and can lead to tissue damage [15]. In contrast to increased neutrophils, lymphocytes decrease during sepsis due to excessive apoptosis [14]. Monocytes, on the other hand, undergo some morphological changes and contribute to cytokine secretion [16]. These changes occurring in sepsis increase the inflammatory response, activate the coagulation cascade and lead to endothelial damage, resulting in platelet activation and aggregation [17]. All these markers can be easily obtained with a CBC and can provide important information regarding the body’s inflammatory status, immune function and changes in disease condition. In addition, these markers may provide an additional contribution to understanding the complex pathophysiological processes of sepsis and aid risk assessment in patients. However, as mentioned above, while some of these markers increase during the inflammatory process, others decrease. For all these reasons, a single hematological biomarker is insufficient to reflect the complex pathophysiological process of sepsis. To solve this problem, some inexpensive and easily obtainable combined biomarkers that can be derived from a CBC have been introduced into clinical use and been investigated in many studies.

Recent additions to the array of available combined markers are the SII, the SIRI and the AISI. Studies have shown that they may be more representative of overall body inflammation and metabolic abnormalities and better reflect a dysregulated immune response when compared with conventional inflammatory markers. Increased levels of these markers were found to be linked with poor prognosis in sepsis [11,13,18,19,20]. In line with previous evidence, we also found that the SII, the SIRI and the AISI were significantly higher in non-survivor sepsis patients. Based on all the findings, it may be stated that these new combined markers are effective in predicting poor prognosis in patients with sepsis.

CRP and albumin are traditional biochemical markers of systemic inflammation. CRP is a positive acute-phase protein indicating tissue damage and infection, while albumin is a negative acute-phase protein reflecting the body’s nutritional status [21,22]. Studies have shown that increased CRP and decreased albumin level are associated with increased mortality and poor prognosis in patients with sepsis [22]. The IPI is a novel combined inflammatory marker including CRP and albumin in addition to the neutrophil and lymphocyte counts in the CBC that has been developed to evaluate the immunological and inflammatory status of patients [23]. The combination of NLR with CRP and albumin may reflect the inflammatory process in more detail; indeed, its clinical importance has been confirmed in various diseases where this marker has been found to be associated with poor prognosis [12,23,24,25,26]. However, no studies have investigated the impact of the IPI on prognosis prediction in patients with sepsis. In the present study, it was found that the IPI was significantly higher in non-survivor sepsis patients. Therefore, it may be concluded that the IPI is a simple and easily obtainable parameter that can help physicians predict poor prognosis in sepsis patients who may be at high risk of mortality.

Furthermore, in our study, we compared the diagnostic value of traditional and novel combined inflammatory parameters in predicting in-hospital mortality in patients with sepsis. We found that the AUC of the IPI was significantly higher than those of the SII and the AISI and tended to be higher than that of the SIRI for predicting in-hospital mortality. Furthermore, the IPI was independently associated with in-hospital mortality in the regression analysis. These results suggest that the IPI has moderately higher discriminatory ability than the SII and the AISI and mildly higher discriminatory ability than the SIRI to predict poor prognosis in patients with sepsis.

The GCS is a commonly used score to evaluate the level of consciousness in critically ill patients and provides an objective measure of neurological function [27]. Previous studies showed that a decreased GCS score at the time of admission was a predictor of mortality in patients with sepsis [28,29,30]. We also found that the GCS score on admission was significantly lower in non-survivor patients and that it was an independent predictor of mortality. These findings confirm that the GCS is a good prognostic marker in sepsis patients. In addition, we found a weak negative correlation between the GCS score and the IPI in the present study. Therefore, it may be suggested that a more widespread inflammatory response is triggered in sepsis patients with more severe neurological deficit. However, it should also be noted that this causality cannot be inferred solely based on correlation analysis.

### Limitations

Our study had some limitations. The first and most important limitation is the small number of patients, along with the single-center and retrospective nature of the study design. Second, we investigated only in-hospital mortality and not long-term mortality. Third, we measured inflammatory markers only at the time of hospital admission and did not evaluate them with serial measurements. Fourth, due to the retrospective nature of our study, we were unable to access the Sequential Organ Failure Assessment (SOFA) score, quick SOFA and acute physiology and chronic health evaluation-II (APACHE-II) scores; examining these scores, evaluating their relationship with the IPI and understanding their impact on in-hospital mortality prediction could have provided additional insights. Finally, our study lacks external validation, and there may be potential selection bias.

## 5. Conclusions

The IPI is a novel combined inflammatory marker that can be easily obtained from laboratory parameters and may be useful in predicting poor prognosis in high-risk sepsis patients. In the present study, we found that the IPI had moderately higher discriminatory ability than the SII and the AISI in this retrospective sample of patients with sepsis, which requires external validation. However, it should be noted that our results are preliminary and should be confirmed in further prospective studies with a larger sample size.

## Figures and Tables

**Table 1:** Comparison of baseline characteristics of study groups.

	Survivors (n = 81)	Non-Survivors (n = 76)	X^2^ or z Value	*p*
Age, years	65 (46–73)	68 (50–76)	−1.323	0.186
Gender, male (%)	40 (49.4)	39 (51.3)	0.059	0.809
HT (%)	18 (22.2)	11 (14.5)	1.563	0.211
DM (%)	13 (16.0)	15 (19.7)	0.364	0.546
Previous history of CAD (%)	6 (7.4)	9 (11.8)	0.892	0.345
COPD (%)	3 (6)	1 (2.1)	0.960	0.617
ARF on admission (%)	13 (16)	12 (15.8)	0.002	0.965
Previous history of CRF (%)	12 (14.8)	7 (9.2)	1.158	0.282
Hemodialysis requirement (%)	21 (25.9)	28 (36.8)	2.176	0.140
Tracheostomy (%)	9 (11.1)	9 (11.8)	−0.021	0.886
Admission GCS	15 (13–15)	12 (10–15)	−4.649	<0.001
Duration of ICU, days	9 (3–19)	10 (5–17)	−0.697	0.486

HT: hypertension; DM: diabetes mellitus; CAD: coronary artery disease; COPD: chronic obstructive pulmonary disease; ARF: acute renal failure; CRF: chronic renal failure; GCS: Glasgow Coma Scale; ICU: intensive care unit.

**Table 2:** Comparison of laboratory parameters of study groups.

	Survivors (n = 81)	Non-Survivors (n = 76)	t or z Value	*p*
Glucose, mg/dL	139 (104–221)	149 (102–213)	−0.249	0.803
BUN, mg/dL	70 (41–111)	82 (53–133)	−1.723	0.085
Creatinine, mg/dL	1.6 (1.0–2.6)	1.8 (1.1–3.2)	−0.618	0.536
pH	7.3 ± 0.1	7.3 ± 0.2	−0.068	0.946
Lactate, mmol/L	2.8 (1.9–5.0)	2.7 (1.9–4.2)	−0.020	0.984
Hemoglobin, g/dL	11.1 ± 2.6	11.4 ± 2.9	0.708	0.480
Platelet, ×10^3^/μL	237 (147–326)	320 (155–312)	−0.541	0.589
WBC, ×10^3^/μL	12.2 (8.2–16.3)	14.2 (10.1–17.1)	−2.000	0.045
Neutrophil, ×10^3^/μL	9.3 (5.4–13.4)	12.2 (7.4–14.8)	−2.459	0.014
Lymphocyte, ×10^3^/μL	1.7 (1.1–2.3)	1.2 (0.7–1.9)	−3.162	0.002
Monocyte, ×10^3^/μL	0.56 (0.33–0.76)	0.60 (0.45–0.83)	−1.363	0.173
MPV, fL	10.7 ± 1.2	10.7 ± 1.1	−0.096	0.924
MCV, fL	84.8 ± 7.4	83.4 ± 7.3	−1.177	0.241
CRP, mg/dL	7.9 (1.7–14.4)	16.1 (9.4–23.7)	−4.104	<0.001
Albumin, g/dL	3.1 ± 0.7	2.9 ± 0.7	−1.674	0.096
SII	1259 (548–2436)	2149 (1207–4032)	−3.123	0.002
SIRI	2.5 (0.8–7.2)	5.9 (3.0–14.6)	−3.911	<0.001
AISI	527 (159–1436)	1252 (503–3160)	−3.092	0.002
IPI	11.7 (1.5–35.2)	60.5 (14.6–128.8)	−5.072	<0.001

BUN: blood urea nitrogen; WBC: white blood cell; MPV: mean platelet volume; MCV: mean corpuscular volume; CRP: C-reactive protein; SII: systemic immune-inflammation index; SIRI: systemic inflammatory response index; AISI: aggregate index of systemic inflammation; IPI: inflammatory prognostic index.

**Table 3:** Independent predictors of in-hospital mortality.

	B	SE	Wald	OR	95% CI	*p*
Admission GCS	−0.270	0.075	12.916	0.764	0.659–0.885	<0.001
IPI	0.007	0.002	7.569	1.007	1.002–1.012	0.006

Entered variables: Age, gender, HT, DM, CAD, ARF, hemodialysis requirement, admission GCS, creatinine, hemoglobin and IPI.

## Data Availability

The data presented in this study are available upon request from the corresponding author. The data are not publicly available due to the arrangements made by the Ethics Committee.

## Abbreviations

The following abbreviations are used in this manuscript:

SII	Systemic immune-inflammation index
SIRI	Systemic inflammatory response index
AISI	Aggregate index of systemic inflammation
IPI	Inflammatory prognostic index
GCS	Glasgow Coma Scale
AUC	Area under the curve
ROC	Receiver operating characteristic
